# Heat acclimation improves exercise performance in hot conditions and increases heat shock protein 70 and 90 of skeletal muscles in Thoroughbred horses

**DOI:** 10.14814/phy2.16083

**Published:** 2024-05-24

**Authors:** Yusaku Ebisuda, Kazutaka Mukai, Yuji Takahashi, Toshinobu Yoshida, Tsubasa Matsuhashi, Aoto Kawano, Hirofumi Miyata, Masayoshi Kuwahara, Hajime Ohmura

**Affiliations:** ^1^ Sports Science Division Equine Research Institute, Japan Racing Association Shimotsuke Japan; ^2^ Department of Biological Sciences, Graduate School of Sciences and Technology for Innovation Yamaguchi University Yamaguchi Japan; ^3^ Department of Veterinary Pathophysiology and Animal Health, Graduate School of Agricultural and Life Sciences The University of Tokyo Tokyo Japan

**Keywords:** exercise performance, heat acclimation, horse, skeletal muscle

## Abstract

This study aimed to determine whether heat acclimation could induce adaptations in exercise performance, thermoregulation, and the expression of proteins associated with heat stress in the skeletal muscles of Thoroughbreds. Thirteen trained Thoroughbreds performed 3 weeks of training protocols, consisting of cantering at 90% maximal oxygen consumption (*V*O_2max_) for 2 min 2 days/week and cantering at 7 m/s for 3 min 1 day/week, followed by a 20‐min walk in either a control group (CON; Wet Bulb Globe Temperature [WBGT] 12–13°C; *n* = 6) or a heat acclimation group (HA; WBGT 29–30°C; *n* = 7). Before and after heat acclimation, standardized exercise tests (SET) were conducted, cantering at 7 m/s for 90 s and at 115% *V*O_2max_ until fatigue in hot conditions. Increases in run time (*p* = 0.0301), peak cardiac output (*p* = 0.0248), and peak stroke volume (*p* = 0.0113) were greater in HA than in CON. Pulmonary artery temperature at 7 m/s was lower in HA than in CON (*p* = 0.0332). The expression of heat shock protein 70 (*p* = 0.0201) and 90 (*p* = 0.0167) increased in HA, but not in CON. These results suggest that heat acclimation elicits improvements in exercise performance and thermoregulation under hot conditions, with a protective adaptation to heat stress in equine skeletal muscles.

## 1 INTRODUCTION

The combination of environmental heat stress and exercise increases physiological strain, including excessive elevation of core and skin temperatures (Arngrimsson et al., 2003; Ely et al., 2010; Nybo et al., 2001; Nybo & Nielsen, 2001; Parkin et al., 1999; Periard et al., 2011; Tucker et al., 2004), increased cardiovascular strain (e.g., elevated heart rate and reduced stroke volume) (Nybo & Nielsen, 2001; Periard et al., 2011; Rowell et al., 1966; Rowell et al., 1969), and decreased aerobic capacity in humans (Arngrimsson et al., 2003; No & Kwak, 2016; Nybo et al., 2001). Earlier research in humans has suggested that these physiological strains can be risk factors for impaired exercise performance (Ely et al., 2010; MacDougall et al., 1974; Parkin et al., 1999; Periard et al., 2011; Tucker et al., 2004) and exertional heat illness (Casa et al., 2015; Cooper et al., 2016; Periard et al., 2021). The influence of environmental heat stress was particularly pronounced during endurance events. Several studies have demonstrated the efficacy of heat acclimation in humans as a countermeasure for mitigating the impact of environmental heat stress on athletes. Heat acclimation is the process of repeated exposure to a hot environment that induces positive physiological adaptations, including decreased body temperature, lowered heart rate, and increased stroke volume (Febbraio et al., 1994; Keiser et al., 2015; Lorenzo et al., 2010; Sawka et al., 1985). Furthermore, heat acclimation can induce adaptation at the cellular and physiological levels (Kuennen et al., 2011). Several in vitro studies have demonstrated that repeated exposure to heat stress induces the upregulation of heat shock proteins (HSPs), a family of proteins that plays important roles in protecting against cellular injury related to heat stress, and proteins related to mitochondrial biogenesis (Liu & Brooks, 2012; Patton et al., 2018).

Thoroughbred horses have a high capacity for energy production and produce large amounts of heat during strenuous exercise. Therefore, if thermoregulatory mechanisms fail to dissipate the accumulated heat, the body temperature increases by approximately 1°C per min in horses (Hodgson, 2014). Furthermore, the heat dissipation efficiency is considerably reduced at high ambient temperatures and humidity (Brownlow et al., 2016; Hodgson, 2014). Strenuous exercise in such situations causes a remarkable increase in the body temperature of horses, even when the exercise duration is short (Ebisuda, Mukai, Takahashi, & Ohmura, 2023). In fact, previous research in Thoroughbred horses has demonstrated a strong relationship between the increased risk of post‐race exertional heat illness and elevation in the wet bulb globe temperature (WBGT) (Takahashi & Takahashi, 2020). Therefore, there is a continuous need for Thoroughbred horses to decrease their physiological strain and exertional heat illness in hot environments. Several reports on horses have shown that daily heat exposure has beneficial effects on physiological responses, similar to those in humans (Geor et al., 1996; Geor et al., 2000; Kang et al., 2023; Marlin et al., 1999). In particular, Marlin et al. (1999) demonstrated that 15 consecutive days of training consisting of various intensity levels (ranging from 30% to 95% *V*O_2max_) for 80 min at 30°C and 80% relative humidity (RH) induced heat acclimation adaptations, including decreased body temperature and improved exercise tolerance under hot conditions. However, earlier studies have evaluated the effect of long‐duration exercise for consecutive days in a small proportion of high‐intensity exercise; hence, there is a concern that high‐intensity and long‐duration training in hot conditions for consecutive days leads to a risk of excessive stress. Duvnjak‐Zaknich et al. (2019) demonstrated that both continuous heat exposure (8 sessions over 8 days) and intermittent heat exposure (8 sessions over 15 days) elicited improvements in exercise performance in team sports athletes. In addition, Houmard et al. (1990) demonstrated that moderate‐intensity (75% *V*O_2max_) and short‐duration (30–35 min) exercise in a hot environment induced heat acclimation, including reductions in core temperature and energy expenditure during a heat tolerance test, similar to low‐intensity (50% *V*O_2max_) and long‐duration (60 min) exercise in trained human subjects. Therefore, we hypothesized that an intermittent heat acclimation protocol combined with high‐intensity and short‐duration training in hot conditions would induce physiological adaptation to heat stress with a reduced risk of overtraining in Thoroughbred horses. In addition, high‐intensity training can induce cellular adaptations in equine skeletal muscles (Kitaoka et al., 2012). However, there is insufficient information regarding the effects of high‐intensity training combined with heat exposure on skeletal muscle adaptations in Thoroughbred horses. In fact, we previously reported an augmentation in mRNA expression of HSP‐70 and peroxisome proliferator‐activated receptor γ coactivator 1α (PGC‐1α), a master regulator of mitochondrial biogenesis, in response to a single bout of exercise in hot conditions (Ebisuda, Mukai, Takahashi, Yoshida, et al., 2023). These adaptations have the potential to improve skeletal muscle metabolism and increase thermal tolerance, leading to beneficial effects in improving aerobic exercise performance in heat and preventing exertional heat illnesses. A better understanding of heat acclimation can help develop effective training strategies for racing in hot environments and contribute to improving the welfare of Thoroughbred racehorses.

The purpose of this study was to test the hypothesis that training Thoroughbred horses 3 sessions per week for three weeks in a hot environment could induce greater adaptations in exercise performance, thermoregulatory response, and proteins related to heat stress and mitochondrial biogenesis in skeletal muscles compared with training in a cool environment.

## 2 MATERIALS AND METHODS

### 2.1 Ethics statement

The study protocol was approved by the Animal Welfare and Ethics Committee of the Equine Research Institute of the Japan Racing Association (accession number 21–1). All incisions for catheter placement and muscle biopsies were performed under local anesthesia using lidocaine to minimize animal suffering.

### 2.2 Animals

Although 14 Thoroughbred horses were trained in this study, one horse was excluded from the analysis because of lameness during the experimental period. Thirteen healthy Thoroughbred horses (six castrated males and seven females; age, 4.6 ± 1.6 [mean ± SD] years; body weight, 498 ± 53 kg at the onset of the study) were included in this study. The horses underwent a preliminary surgery to move the left carotid artery from the carotid sheath to a subcutaneous location to facilitate arterial catheterization. Horses exercised 2 days/week on a treadmill under cool conditions (WBGT, 15°C) and walked for 1 h/day in a walker on the other 5 days for 4 weeks prior to the experiment. The horses performed a training program composed of walking at 1.7 m/s for 30 min in a walker, trotting at 4 m/s for 5 min, and cantering at 7 m/s for 2 min and 10 m/s for 2 min on a treadmill inclined at 6%, followed by walking at 1.7 m/s for 30 min in a walker. After the preliminary training period, the horses performed the incremental exercise test in a cool condition (WBGT, 15°C) to measure their maximal oxygen consumption (*V*O_2max_) and the speed eliciting *V*O_2max_. Following a warm‐up at 4 m/s for 3 min, each horse exercised for 2 min at 1.7, 4, 6, 8, 10, 12, and 13 m/s on a 6% inclined treadmill until they could not maintain their position in front of the treadmill with human encouragement. This was defined as exhaustion. *V*O_2_ was calculated for the final 30 s of each step.

### 2.3 Experimental design

This study was conducted during winter (January to March), when the horses were not acclimated to hot conditions. The horses performed a standardized exercise test (SET) in hot condition (ambient temperature, 37.2 ± 0.9°C; RH, 35.0 ± 3.1%; WBGT, 29.5 ± 0.7°C) before (pre) and after (post) the heat acclimation period. After all catheters were placed, a heart rate monitor (S810, Polar, Kempele, Finland) was attached, and each horse was warmed up by trotting at 4 m/s for 3 min. Horses wore an open‐flow mask to measure *V*O_2_ and exercised at 6% inclination for 90 s each at 4 m/s and 7 m/s followed by cantering at a speed eliciting 115% of *V*O_2max_ in hot conditions until exhaustion. Run time to exhaustion was measured using a stopwatch. Heart rate was recorded using a commercial heart rate monitor (S810, Polar, Kempele, Finland), and the mean heart rate was calculated for the final 30 s of each step. Body weight was measured before the SET using a weight scale (RT‐1C, Kubota, Osaka, Japan). The horses were divided into control (CON, *n* = 6) and heat acclimation (HA, *n* = 7) groups to match the pre‐acclimation runtime. Three weeks of a heat acclimation program consisted of high‐intensity exercise twice weekly (walking at 1.7 m/s for 1 min, trotting at 4 m/s for 5 min, and cantering at 7 m/s for 2 min and at the speed eliciting 90% *V*O_2max_ for 2 min, followed by walking at 1.7 m/s for 20 min) and moderate intensity exercise once weekly (walking at 1.7 m/s for 1 min, trotting at 4 m/s for 6 min, and cantering at 7 m/s for 3 min, followed by walking at 1.7 m/s for 20 min) on a 6% inclined treadmill. All horses walked for 30 min on an outdoor walker prior to treadmill exercise. Table 1 shows the environmental conditions during treadmill training in the CON and HA groups. Room temperature and RH were controlled using air conditioners (RAS‐AP140DG4, Hitachi, Tokyo, Japan), oil heaters (HPS360, Orion, Nagano, Japan), and misting fans (HW‐26MC02, SIS, Ube, Japan). Ambient temperature, RH, and WBGT were measured using a portable monitoring device (WBGT‐213B, Kyoto Electronics Manufacturing, Kyoto, Japan). Post‐SET was completed within 1 week of the final training session.

**TABLE 1:** Environmental conditions during treadmill training in the control (CON) and heat acclimation (HA) groups.

	CON (*n* = 6)	HA (*n* = 7)
WBGT (°C)	14.3 ± 1.4	29.8 ± 0.8
Ambient temperature (°C)	20.2 ± 1.9	37.6 ± 1.8
Relative humidity (%)	31.5 ± 8.2	35.3 ± 3.4

*Note*: Values represent the mean ± SD. WBGT, Wet Bulb Globe Temperature.

### 2.4 Oxygen consumption

The procedure for measuring oxygen consumption has been described previously (Birks et al., 2019; Ebisuda, Mukai, Takahashi, & Ohmura, 2023; Jones et al., 1989; Mukai et al., 2020). The horses wore a 25‐cm‐diameter open‐flow mask on a treadmill with rheostat‐controlled blower drawing air. Air passed through a 25‐cm‐diameter tubing and across a pneumotachograph (LF‐150B, Vise Medical, Chiba, Japan) connected to a differential pressure transducer (TF‐5, Vise Medical, Chiba, Japan). This ensured that the bias flows during measurements were identical to those used during calibration. Oxygen and CO_2_ concentrations were measured using an O_2_ and CO_2_ analyzer (O_2_, FC‐10 Oxygen Analyzer, Sable Systems International, NV; CO_2_, MG‐360, Vise Medical, Chiba, Japan), and calibrations were performed to calculate the rates of O_2_ consumption and CO_2_ production via electronic mass flow meters (CR‐300, Kofloc, Kyoto, Japan) and using the N_2_‐dilution/CO_2_‐addition mass‐balance technique (Fedak et al., 1981). The gas analyzer and mass flow meter outputs were recorded on a personal computer and analyzed using commercial hardware and software (DI‐720 and Windaq Pro+; DATAQ, Akron, OH, USA).

### 2.5 Blood sampling

An 18‐gauge, 5.1‐cm catheter (SR‐FF1851, Terumo, Tokyo, Japan) was placed in the left carotid artery, and an 8 F, 10‐cm introducer (RS‐A80K10S, Terumo, Tokyo, Japan) was placed in the right jugular vein under local anesthesia (2% xylocaine). In the right jugular vein, a Swan‐Ganz catheter (SP5107U; Becton, Dickinson and Company, Franklin Lakes, NJ, USA) was inserted through the introducer, and the tip of the catheter was positioned in the pulmonary artery, as confirmed by measuring the pressure waveform at its distal tip using a pressure transducer (P23XL, Becton, Dickinson and Company). Arterial and mixed venous blood samples were collected simultaneously during the final 30 s of 7 m/s cantering and every 40 s until exhaustion during the supramaximal exercise. Samples were analyzed using a blood gas analyzer (ABL800 FLEX, Radiometer, Copenhagen, Denmark), and O_2_ saturation and O_2_ concentration were measured using a hemoximeter (ABL80 FLEX‐CO‐OX, Radiometer, Copenhagen, Denmark) set to its equine algorithm. Pulmonary artery temperature (*T*_PA_) during exercise was measured at each blood sampling using a thermistor of the Swan–Ganz catheter connected to a cardiac output computer (COM‐2, Baxter International, Deerfield, IL, USA) and was used to correct the blood gas measurements. After measuring blood gases and oximetry, blood samples were centrifuged (AX‐511, Tomy Industrial, Tokyo, Japan) at 1740 × *g* for 10 min to measure plasma lactate concentration using a lactate analyzer (Biosen S‐Line; EKF‐diagnostic GmbH, Barleben, Germany). Cardiac output was calculated as oxygen consumption divided by the difference in arterial‐mixed venous oxygen concentration difference (Fick principle), and cardiac stroke volume was calculated as cardiac output divided by heart rate.

### 2.6 Muscle biopsy

In each SET, muscle samples (~50 mg wet weight) were obtained from the same area (the two sampling points were approximately 2 cm apart) at the midsection of the *gluteus medius* muscle and from the same depth (5 cm below the skin surface) using needle biopsy under local anesthesia (Lidocaine, Fujisawa Pharmaceutical, Osaka, Japan) before exercise. All muscle samples were immediately frozen in liquid nitrogen and stored at −80°C until analysis.

### 2.7 Western blotting

The *gluteus medius* muscle sample was homogenized in lysis buffer (1% Triton x‐100, 50 mmol/L Tris–HCl, 1 mM EDTA, 1 mM EGTA, 50 mM NaF, 10 mM sodium β‐glycerol phosphate, 5 mM sodium pyrophosphate, 2 mM DTT, 1 mM Na orthovanadate, 1 mM PMSF, and 10 μg/μL of each aprotinin, leupeptin, and pepstatin A) supplemented with protease inhibitor cocktail (Halt, #1860932, Thermo Scientific, Waltham, MA) and phosphatase inhibitor mixture (PhosSTOP, #04906837001, Roche Diagnostics, Mannheim, Germany). The total protein content of the samples was quantified using the Bradford protein assay (Quick Start, #500–0205, Bio‐Rad Laboratories, Hercules, CA, USA) at a wavelength of 595 nm using a microplate reader (Spark, Tecan Group, Männedorf, Switzerland). Equal amounts of protein were loaded onto 10% stain‐free gels (TGX Stain‐Free Fast Cast Acrylamide Kit, #1610183, Bio‐Rad Laboratories, Hercules, CA, USA) and separated by electrophoresis at a constant voltage of 200 V for 30 min. After loading, the stain‐free gels were activated with UV light. Proteins were transferred to 0.2 μm polyvinylidene difluoride membranes using the Trans‐Blot Turbo Transfer System (Bio‐Rad Laboratories, Hercules, CA). The membranes were blocked in 5% BSA in Tris‐buffered saline containing 0.1% Tween 20 (TBS‐T) for 1 h at room temperature, followed by overnight incubation at 4°C with the following primary antibodies: 70‐kDa heat shock protein (HSP‐70; ab79852, Abcam, Cambridge, UK), 90‐kDa heat shock protein (HSP‐90; ab13495, Abcam, Cambridge, UK), peroxisome proliferator‐activated receptor γ coactivator 1α (PGC‐1α; ab54481 Abcam, Cambridge, UK), and cytochrome C oxidase subunit 4 (COX4; #4844, Cell Signaling Technology, Danvers, MA, USA). After washing with TBS‐T, secondary antibody incubation was performed using an appropriate anti‐rabbit antibody (#7074, Cell Signaling Technology, Danvers, MA, USA) for 1 h at room temperature. The membranes were washed again with TBS‐T and developed using chemiluminescent reagents (Clarity Western ECL Substrate, #170–5061, Bio‐Rad Laboratories, Hercules, CA, USA). Proteins were detected using ChemiDoc XRS+ (Bio‐Rad Laboratories, Hercules, CA, USA). Band intensity was quantified by total protein normalization using commercial software (Image Lab 5.1, Bio‐Rad Laboratories, Hercules, CA, USA).

### 2.8 Metabolic enzyme activity

The procedures for succinate dehydrogenase (SDH) and β‐3‐hydroxy acyl‐CoA dehydrogenase (HAD) activity have been previously described (Eto et al., 2004). Muscle samples (10–20 mg) from each horse were prepared to measure muscle enzyme activity. The samples were homogenized in ice‐cold 33.3 mM phosphate buffer (pH 7.4). SDH activity of the homogenate was measured using the technique described by Cooperstein et al. (1950). Another piece of muscle (10–20 mg) was homogenized in an ice‐cold homogenization medium containing 175 mM KCl, 10 mM glutathione, and 2 mM EDTA. HAD activity was determined as described by Bass et al. (1969).

### 2.9 Statistical procedures

All data are presented as mean ± standard deviation (SD). After the acclimation period, within‐subject changes in physiological variables were analyzed using Student's *t*‐test for differences between groups. Protein expression was analyzed using a mixed model with time and group as fixed effects and individual horses as random effects. When a significant main effect or interaction between the main effects was observed, Tukey's tests were used as post hoc tests. For all analyses, statistical significance was defined as *p* < 0.05. Statistical software (JMP 16.2.0, SAS Institute Inc., Cary, NC, USA) was used for all data analyses.

## 3 RESULTS

**FIGURE 1:**
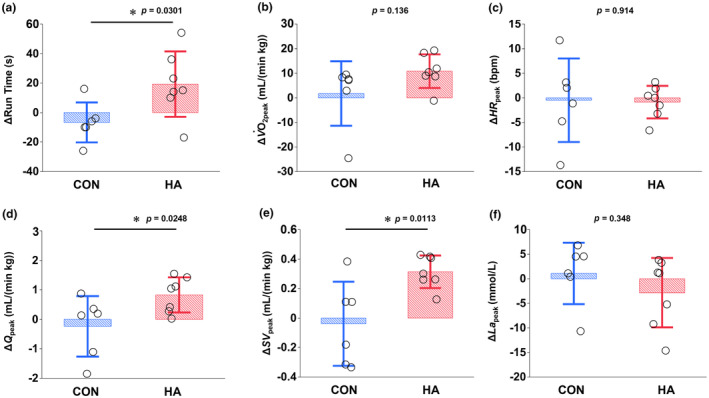
Changes in run time (*p* = 0.0301; (a), peak oxygen consumption (*V*O_2peak_, *p* = 0.136; (b), peak heart rate (*HR*_peak_, *p* = 0.914; (c), peak cardiac output (*Q*_peak_, *p* = 0.0248; (d), peak stroke volume (*SV*_peak_, *p* = 0.0113; (e), and peak plasma lactate concentration (*La*_peak_, *p* = 0.348; (f) during standardized exercise test after 3 weeks of training either in cool (CON; *n* = 6) or hot (HA; *n* = 7) conditions. Values represent the mean ± SD. *Significant differences between groups (*p* < 0.05).

**FIGURE 2:**
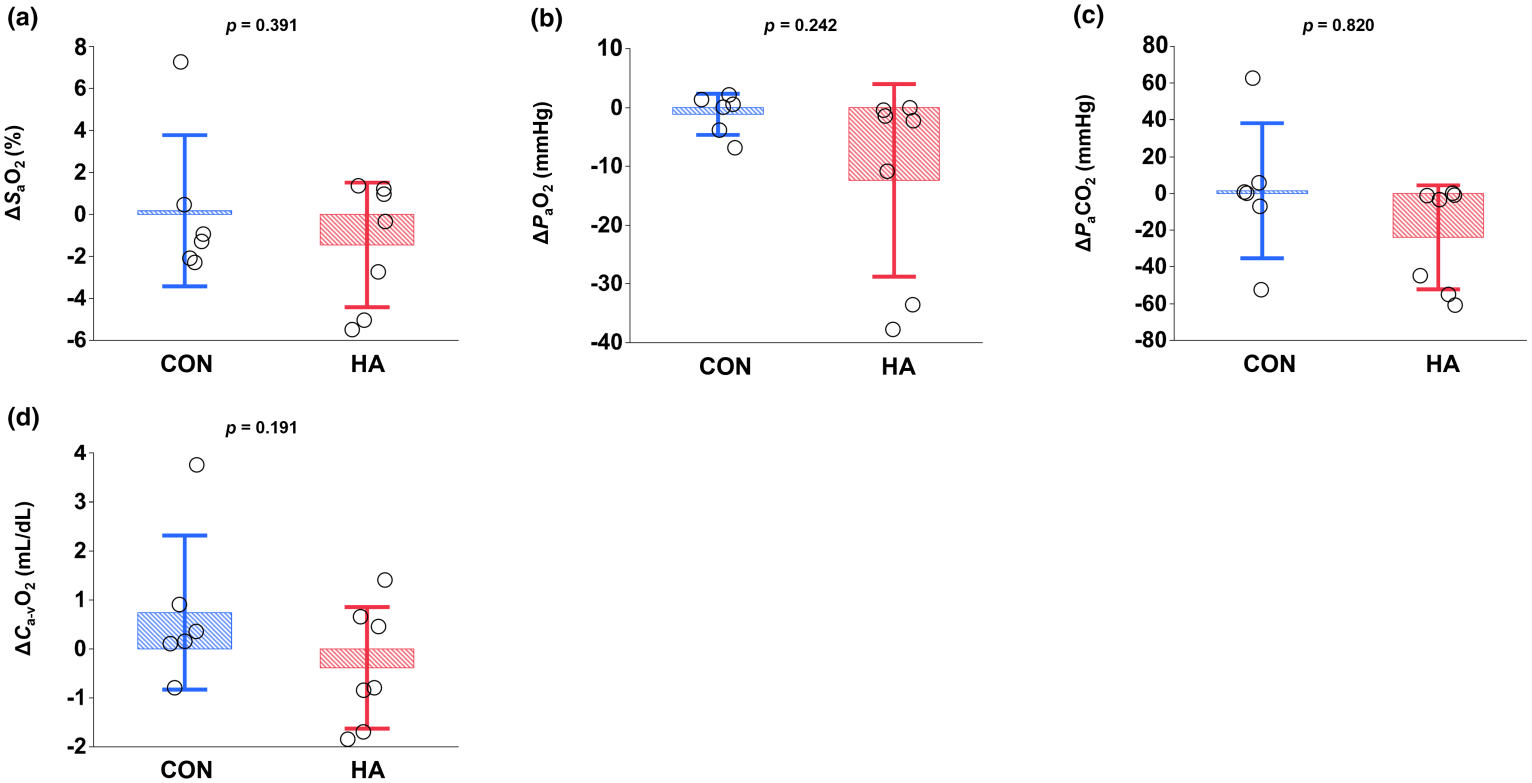
Changes in arterial O_2_ saturation (*S*_a_O_2_, *p* = 0.0391; (a), arterial O_2_ partial pressure (*P*_a_O_2_, *p* = 0.242; (b), arterial CO_2_ partial pressure (*P*_a_CO_2_, *p* = 0.820; (c), and arteria‐mixed venous O_2_ difference (*C*_a‐v_O_2_, *p* = 0.191; (d) during the standardized exercise test after 3 weeks of training either in cool (CON; *n* = 6) or hot (HA; *n* = 7) conditions. Values represent the mean ± SD.

**FIGURE 3:**
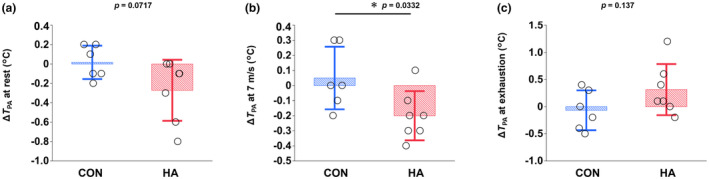
Changes in pulmonary artery temperature (*T*_PA_) at rest (*p* = 0.0717; a), 7 m/s (*p* = 0.0332; b), and exhaustion (*p* = 0.137; c) during the standardized exercise test after 3 weeks of training in either cool (CON; *n* = 6) or hot (HA; *n* = 7) conditions. Values are mean ± SD. *Significant differences between groups (*p* < 0.05).

**FIGURE 4:**
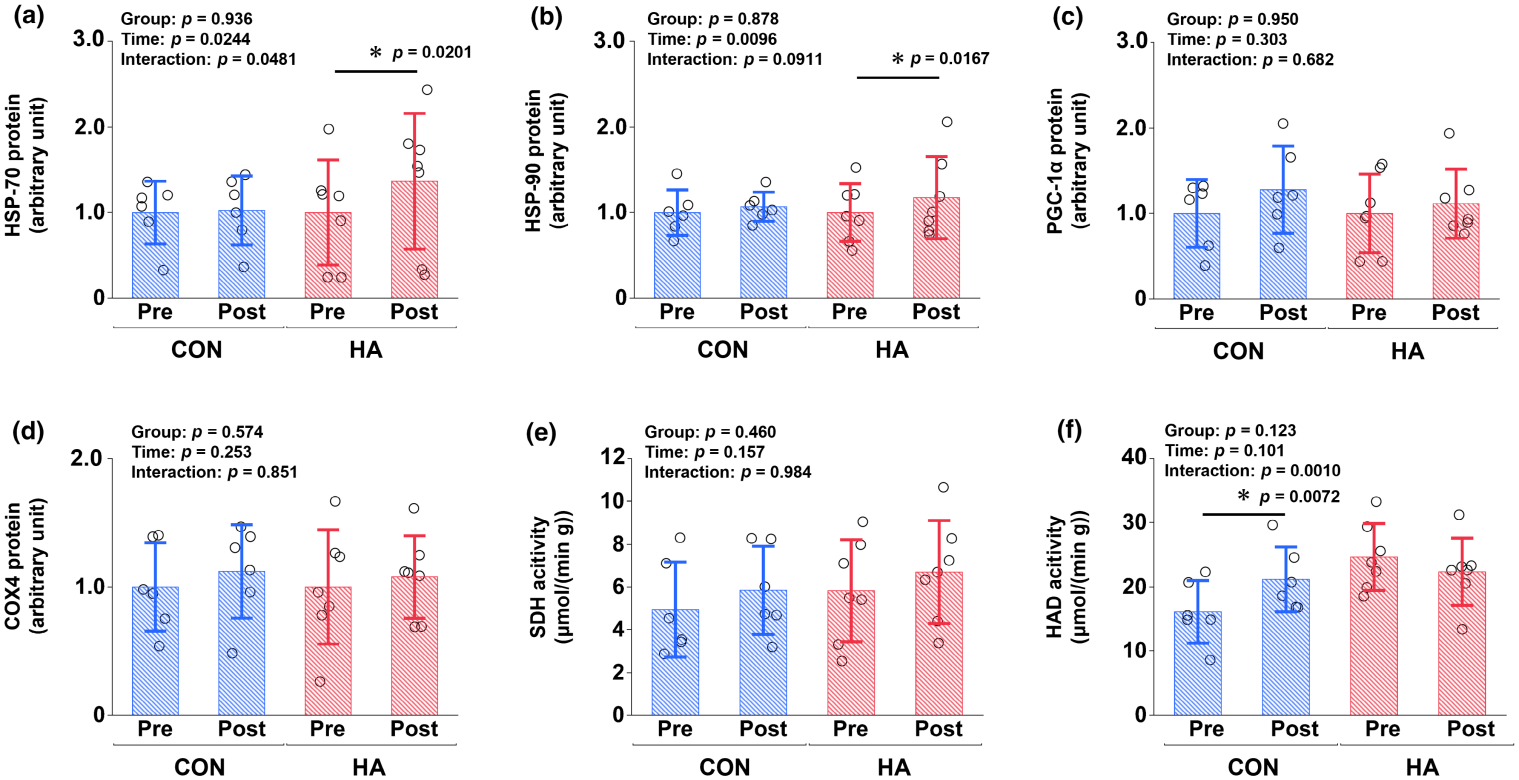
Heat shock protein (HSP)‐70 (a), HSP‐90 (b), peroxisome proliferator‐activated receptor γ coactivator 1α (PGC‐1α; c), cytochrome c oxidase 4 (COX4; d) protein content, succinate dehydrogenase (SDH) activity (e), and β‐3‐hydroxy acyl CoA dehydrogenase (HAD) activity (f) before (pre) and after (post) 3 weeks of training either in cool (CON; *n* = 6) or heat (HA; *n* = 7) conditions. Values represent the mean ± SD. *Significant differences between pre‐ and post‐intervention (*p* < 0.05).

The physical and physiological characteristics of the horses were not significantly different between the CON and HA groups in terms of age, body weight, or *V*O_2max_ measured under cool conditions before heat acclimation (Table 2). Table 3 summarizes the parameters of exercise performance, aerobic capacity, blood gas, and pulmonary artery temperature before (pre) and after (post) heat acclimation. After heat acclimation, the change in run time was greater in HA than in CON (CON, −8.1%; HA, +24.5%; *p* = 0.0301; Figure 1). The changes in peak cardiac output (Δ*Q*_peak_; CON, −2.1%; HA, +8.6%; *p* = 0.0248) and peak stroke volume (Δ*SV*_peak_; CON, −2.2%; HA, +9.2%; *p* = 0.0113) after heat acclimation were also greater in HA than in CON, whereas the changes in peak oxygen consumption (Δ*V*O_2peak_; CON, +1.1%; HA, +6.9%; *p* = 0.136), peak heart rate (Δ*HR*_peak_; CON, −0.4%; HA, −0.2%; *p* = 0.914), and peak plasma concentration (Δ*La*_peak_; CON, −2.1%, HA, +2.7%; *p* = 0.348) were not different between the two groups (Figure 1). Blood gas variables, including arterial O_2_ saturation (*S*_a_O_2_), arterial O_2_ partial pressure (*P*_a_O_2_), arterial CO_2_ partial pressure (*P*_a_CO_2_), and arterial‐mixed venous O_2_ concentration difference (*C*_a‐v_O_2_), did not differ between the groups (*S*_a_O_2_, *p* = 0.391; *P*_a_O_2_, *p* = 0.242; *P*_a_CO_2_, *p* = 0.820; *C*_a‐v_O_2_, *p* = 0.191; Figure 2). After heat acclimation, the changes in *T*_PA_ at rest (CON, +0.1%; HA, −0.7%; *p* = 0.0717) and exhaustion (CON, −0.2%; HA, +0.8%; *p* = 0.137) in the SET were not different between the groups, whereas the decrease in *T*_PA_ at 7 m/s was greater in HA than in CON (CON, +0.1%; HA, −0.5%; *p* = 0.0332; Figure 3). HSP‐70 and HSP‐90 protein expression increased after heat acclimation in HA (HSP‐70, *p* = 0.0201; HSP‐90, *p* = 0.0167) but not in CON (HSP‐70, *p* = 0.909; HSP‐90, *p* = 0.820) (Figure 4). In contrast, PGC‐1α protein (*p* = 0.303), COX4 protein (*p* = 0.253), and SDH activity (*p* = 0.157) did not change in either group after heat acclimation. HAD activity increased in CON (*p* = 0.0072) but not in HA (*p* = 0.230).

**TABLE 2:** Characteristic data of the control (CON) and heat acclimation (HA) groups before heat acclimation.

	CON (*n* = 6)	HA (*n* = 7)
Sex	2 castrated males 4 females	4 castrated males 3 females
Age (years)	4.2 ± 1.6	5.0 ± 1.6
Body weight (kg)	485 ± 58	509 ± 50
*V*O_2max_ (mL/(min kg))	177 ± 17	172 ± 20
100% *V*O_2max_ (m/s)	11.8 ± 0.4	11.3 ± 0.9

*Note*: Values represent the mean ± SD. The reported values of maximal oxygen consumption (*V*O_2max_) and speed eliciting 100% *V*O_2max_ were measured under cool conditions (Wet Bulb Globe Temperature [WBGT], 15°C).

**TABLE 3:** Parameters on exercise performance, aerobic capacity, blood gas, and pulmonary artery temperature in standardized exercise test (SET) at pre‐ and post‐heat acclimation.

	CON (*n* = 6)		HA (*n* = 7)		
	Pre	Post	Pre	Post	
Run time (s)	82.2 ± 16.0	75.5 ± 12.1	78.7 ± 16.0	98.0 ± 20.0	
*V*O_2peak_ (mL/(min kg))	158.6 ± 13.0	160.4 ± 14.0	156.4 ± 17.6	167.3 ± 12.7	
*Q*_peak_ (mL/(min kg))	692.6 ± 48.9	678.4 ± 62.5	646.2 ± 64.4	702.0 ± 46.3	
*SV*_peak_ (mL/kg)	3.20 ± 0.40	3.13 ± 0.32	2.98 ± 0.22	3.25 ± 0.17	
*HR*_peak_ (bpm)	217.5 ± 11.5	217.0 ± 8.9	216.6 ± 7.4	215.7 ± 7.7	
*C*_a‐v_O_2_ (mL/dL)	22.9 ± 1.6	23.7 ± 1.1	24.2 ± 1.6	23.8 ± 1.3	
*P*_a_O_2_ (mmHg)	73.9 ± 5.5	73.7 ± 5.4	79.7 ± 6.5	76.3 ± 5.4	
*P*_a_CO_2_ (mmHg)	62.0 ± 4.8	61.8 ± 4.7	60.4 ± 5.9	60.7 ± 5.7	
*S*_a_O_2_ (%)	81.3 ± 4.2	81.4 ± 2.0	84.0 ± 4.9	82.5 ± 4.1	
*La*_peak (mmol/L)_	35.5 ± 8.6	36.4 ± 7.4	30.6 ± 6.6	30.0 ± 6.6	
*T*_PA_ at rest (°C)	37.7 ± 0.3	37.7 ± 0.3	37.7 ± 0.4	37.4 ± 0.3	
*T*_PA_ at 7 m/s (°C)	39.3 ± 0.3	39.3 ± 0.3	39.3 ± 0.5	39.1 ± 0.4	
*T*_PA_ at exhaustion (°C)	41.3 ± 0.2	41.2 ± 0.3	41.1 ± 0.7	41.4 ± 0.5	

*Note*: Values represent the mean ± SD. Run time, peak oxygen consumption (*V*O_2peak_), peak cardiac output (*Q*_peak_), peak stroke volume (*SV*_peak_), peak heart rate (*HR*_peak_), arterial‐mixed venous O_2_ difference (*C*_a‐v_O_2_), arterial O_2_ partial pressure (*P*_a_O_2_), arterial CO_2_ partial pressure (*P*_a_CO_2_), O_2_ saturation (*S*_a_O_2_) at exhaustion, peak plasma lactate concentration (*La*_peak_), and pulmonary artery temperature (*T*_PA_) at rest, 7 m/s, and exhaustion are presented.

## 4 DISCUSSION

**FIGURE 5:**
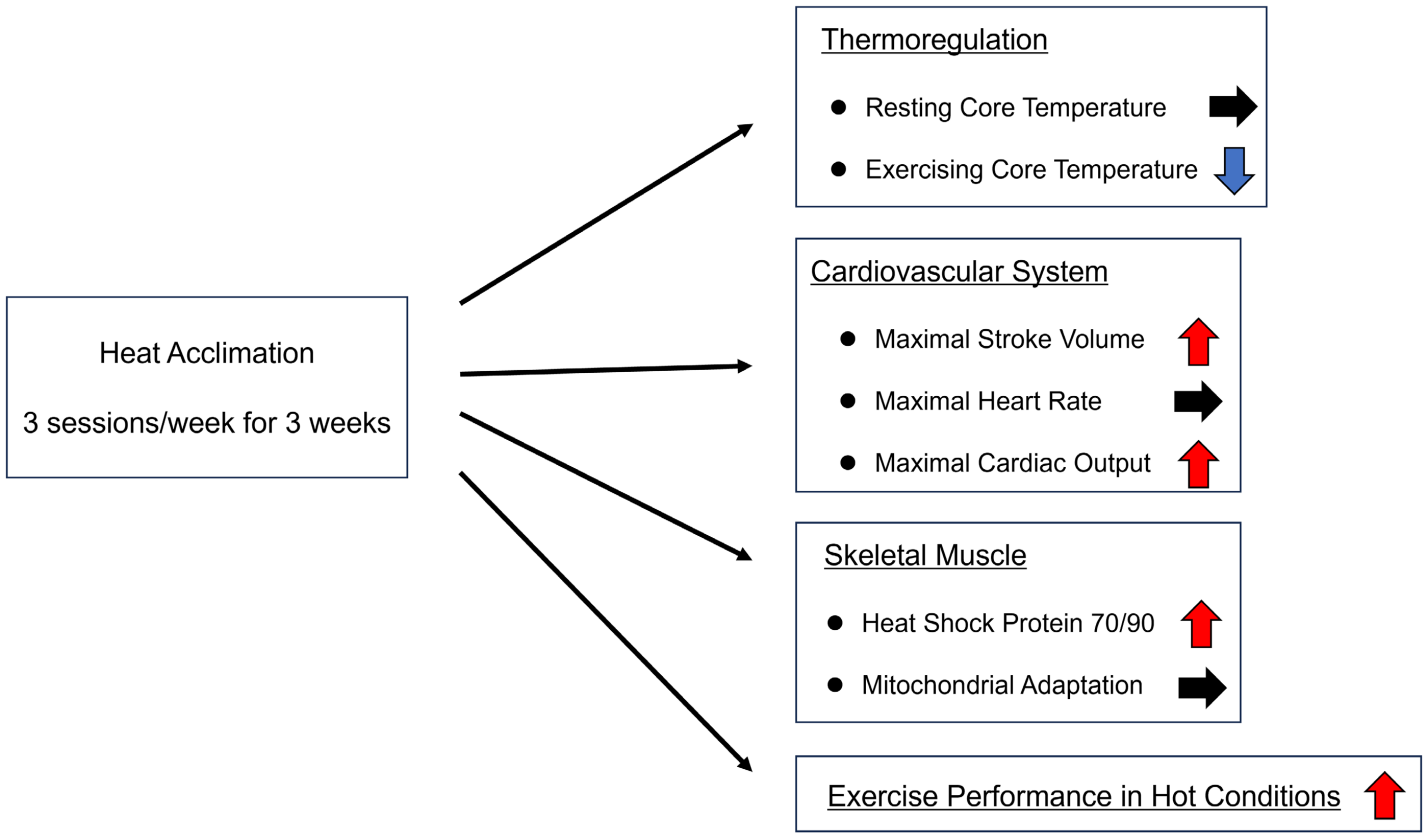
Physiological adaptations after heat acclimation (three sessions/week for three weeks) in Thoroughbred horses.

In this study, we demonstrated that horses trained under hot conditions elicited greater physiological adaptations, including increased run time, cardiac output, stroke volume, and HSP expression in skeletal muscle, and suppressed the elevation of core temperature during submaximal exercise, compared to horses trained under cool conditions (Figure 5). These results suggest that intermittent heat exposure combined with high‐intensity and short‐duration training is sufficient for heat adaptation in Thoroughbred horses.

Several studies in horses have reported that heat acclimation improves exercise performance under hot conditions (Geor et al., 2000; Marlin et al., 1999). Despite our heat acclimation protocol consisting of a shorter duration (30 min) and intermittent sessions (three sessions per week) compared to these studies, we observed that horses trained in hot conditions showed a greater improvement in run time at SET than horses trained in cool conditions. In humans, 7–14 days of consecutive heat exposure is generally recommended to induce heat acclimatization or acclimation; these adaptations require daily heat exposure for 60–90 min combined with exercise (Periard et al., 2021). However, the magnitude of adaptation induced by heat acclimation depends on the intensity, duration, frequency, and number of heat exposures (Périard et al., 2015). Duvnjak‐Zaknich et al. (2019) demonstrated that short‐duration (33–47 min), high‐intensity training (repeated maximal cycling efforts) in heat conducted either intermittently (eight sessions over 15 days) or continuously (eight sessions over eight days) improved repeated‐sprint performance in heat. Although our protocol consisted of short‐duration and intermittent sessions that were modified from the recommended heat acclimation protocols in humans (Periard et al., 2021), high‐intensity training at 90% *V*O_2max_ may have contributed to compensating for these modifications. The important factors that determine the effects of heat acclimation (intensity, duration, frequency, and number of heat exposures) seem to be similar in humans and horses. However, there is insufficient information on the differences in heat acclimation protocols among Thoroughbred horses, and further research on appropriate and effective heat acclimation protocols is needed.

In our study, HA induced a lower *T*_PA_ during submaximal exercise (7 m/s) and higher *Q*_peak_ and *SV*_peak_ under hot conditions than CON. Our finding of decreased core temperature during submaximal exercise is consistent with several reports in horses (Geor et al., 1996; Marlin et al., 1999) and humans (Rowell et al., 1969; Sawka et al., 1985). In horses and humans, the cardiovascular system plays an important role in thermoregulation via cutaneous vasodilation, which increases the blood flow to the skin and dissipates heat from the skin surface to the environment (Hodgson, 2014; Periard et al., 2021). Gonzalez‐Alonso and Calbet (2003) demonstrated that blood flow distribution competed between the skin and skeletal muscles during cycling at 80% peak power output under severe heat stress, which compromised the ability to sustain sufficient blood flow to the locomotive skeletal muscles in humans. In contrast, several studies have demonstrated that chronic heat exposure leads to cardiac and mechanical adaptations in rodents, such as increased myocardial efficiency and enhanced left ventricular compliance (Horowitz et al., 1993; Levy et al., 1997). In humans, 9–12 days of heat acclimation increases stroke volume, cardiac output, and leg blood flow during exercise under hot conditions (Nielsen et al., 1993). Therefore, we speculate that heat acclimation may improve the ability to sustain blood flow to both the skin and skeletal muscles during exercise under hot conditions, which led to decreased core temperature during submaximal exercise and improved exercise performance in this study. Interestingly, despite the longer exercise duration at SET in the HA group than in the CON group, no difference was observed in the peak core temperature at exhaustion. Nielsen et al. (1993) demonstrated that exercise until exhaustion at 60% *V*O_2max_ for 9–12 consecutive days in hot conditions (40°C, 10% RH) did not change the final core temperature at exhaustion (day 1, 39.8 ± 0.13°C; final, 39.7 ± 0.15°C) despite a decrease in the rate of rise of core temperature. Our results are consistent with those of humans, and heat acclimation does not appear to alter the peak core temperature that an individual horse can tolerate during exercise under hot conditions. However, the reduced increase in body temperature during exercise may have contributed to the improved exercise performance.

In humans, several studies have demonstrated that heat acclimation improves *V*O_2max_ under both hot and cool conditions (Benjamin et al., 2019; Lorenzo et al., 2010; Sawka et al., 1985). Particularly, Lorenzo et al. (2010) demonstrated that two 45‐min cycling bouts at 50% *V*O_2max_ under hot conditions (WBGT, 35°C) for 10 consecutive days elicited 8% improvement in *V*O_2max_ in trained athletes under hot conditions. In contrast, Marlin et al. (1999) reported no changes in *V*O_2max_ after heat acclimation consisting of a combination of low‐intensity exercise (30% *V*O_2max_), medium‐intensity exercise (80% *V*O_2max_), and high‐intensity exercise (95% *V*O_2max_) for 15 consecutive days in horses. Consistent with earlier reports on horses (Geor et al., 1996; Marlin et al., 1999), we observed no significant differences between the groups regarding the change in *V*O_2peak_ after heat acclimation. Heat acclimation has been suggested to induce plasma volume expansion, which has the potential to increase *V*O_2max_ in humans (Coyle et al., 1990). In contrast, Marlin et al. (1999) reported no changes in plasma volume after heat acclimation in horses. While the explanation for these contradictory results between humans and horses remains unclear, heat acclimation in horses is less effective in increasing plasma volume than in humans, which may explain the absence of *V*O_2max_ changes in this study.

The novelty of this study lies in determining the effects of heat acclimation on heat stress markers and mitochondrial adaptations in equine skeletal muscles. In humans, heat acclimation induces not only physiological changes that reduce the adverse effects of heat stress but also adaptations at the cellular level, including in peripheral blood mononuclear cells and skeletal muscle tissues (Kuennen et al., 2011; Mang et al., 2021; Maunder et al., 2021). Among the HSP family members, HSP‐70 and ‐90 are considered key factors after a stress encounter because their transcription is highly sensitive to thermal stress (Kruger et al., 2019). Therefore, HSP‐70 and ‐90 may be potential biomarkers reflecting the extent of adaptation to heat stress. Many human studies have reported that heat acclimation increases HSP‐70 and HSP‐90 levels in leukocytes (McClung et al., 2008; Nava & Zuhl, 2020). However, earlier studies on human skeletal muscles have reported inconsistent results regarding HSP upregulation (Mang et al., 2021; Watkins et al., 2008). Watkins et al. (2008) reported no significant increase in HSP‐70 protein expression after 7 days of a heat acclimation protocol consisting of cycling at 75% *V*O_2peak_ for 30 min in a hot environment (39.5°C, 27% RH). In contrast, Mang et al. (2021) demonstrated increased HSP‐70 protein expression after a 10‐day heat acclimation protocol consisting of two 45‐min walking bouts at 30–40% maximal velocity in a hot environment (42–44°C, 30–50% RH). Our results are consistent with those of Mang et al. (2021) and other studies on leukocytes (McClung et al., 2008; Nava & Zuhl, 2020). We found that both basal HSP‐70 and ‐90 protein expression increased after 9 sessions of training in hot conditions over 3 weeks, despite intermittent heat exposure and shorter exposure duration compared to earlier studies in humans (Mang et al., 2021; Watkins et al., 2008). While core body temperature in humans does not exceed 39°C during exercise (Mang et al., 2021; Watkins et al., 2008), core body temperature of Thoroughbred horses sometimes exceeds 41°C after cantering at 10 m/s in a hot environment (WBGT, 29°C) (Ebisuda, Mukai, Takahashi, & Ohmura, 2023). Therefore, higher body temperatures during exercise in horses than in humans may have induced greater HSP upregulation. In addition, high‐intensity exercise causes arterial hypoxemia in Thoroughbred horses, mainly because of the alveolar‐capillary diffusion limitation of O_2_ transport in the lungs, and this phenomenon is more pronounced under hot conditions than under cool conditions (Ebisuda, Mukai, Takahashi, & Ohmura, 2023; Wagner et al., 1989). In vitro research has demonstrated that HSPs, especially HSP‐90, interact with hypoxia‐inducible factor 1α (HIF‐1α), and hypoxia triggers both HSPs and HIF‐1α protein expression (Almgren & Olson, 1999; Kruger et al., 2019; Salgado et al., 2014). We previously demonstrated that acute high‐intensity exercise of cantering at 90% *V*O_2max_ in a hot environment (WBGT, 29.5°C) increased HSP‐70 and HIF‐1α mRNA expression (Ebisuda, Mukai, Takahashi, Yoshida, et al., 2023). Therefore, hypoxemia induced by exercise in a hot environment may have contributed to the increased HSP‐70 and ‐90 protein expressions.

Several studies have demonstrated that chronic heat stress induces mitochondrial adaptation both in vitro and in vivo (Hafen et al., 2018; Liu & Brooks, 2012; Maunder et al., 2021; Patton et al., 2018; Tamura et al., 2014). Tamura et al. (2014) demonstrated that repeated 30‐min heat exposure at 40°C after exercise for 3 weeks (5 days/week) upregulated biomarkers for mitochondrial oxidative capacity (maximal activities of citrate synthase and HAD) and mitochondrial content (COX4 protein) in mouse skeletal muscles. Moreover, Maunder et al. (2021) demonstrated that 15 sessions of endurance training for 3 weeks under hot conditions (33°C, 60% RH), but not in cool conditions (18°C, 60% RH), increased maximal citrate synthase activity in human skeletal muscles. In contrast to these studies, the combination of exercise and heat stress did not induce mitochondrial adaptation, including biomarkers of mitochondrial content and mitochondrial oxidative capacity, in this study. Although the reason for this remains unclear, the stimulus may have been insufficient to induce mitochondrial adaptations, owing to differences in species, exercise duration, exercise mode, ambient temperature, and exposure frequency. However, our previous report showed that PGC‐1α mRNA expression was upregulated 4 h after acute exercise at 90% *V*O_2max_ under hot conditions in Thoroughbred horses (Ebisuda, Mukai, Takahashi, Yoshida, et al., 2023). Repeated transient mRNA upregulation following acute exercise can potentially increase mitochondrial protein levels during training (Hood, 2001; Perry et al., 2010). Although it is unclear why this discordance occurred between exercise‐induced changes in mRNA levels and training‐induced changes in the levels of encoded proteins, similar findings have been documented in human studies (Edgett et al., 2016). Bishop et al. (2023) reported that training‐induced alterations in protein abundance have complex determinants, with transcriptional regulation representing only part of the entire pathway, including post‐transcriptional regulation, translational efficiency, and post‐translational modifications, making it difficult to accurately predict long‐term adaptations from only the results of acute exercise. Although our results demonstrated no additional effects of the combination of exercise and heat stress on mitochondrial adaptation, further studies are needed to better understand the effects and mechanisms of heat acclimation in equine skeletal muscles.

One limitation of this study comprises the inability to rule out the possibility of sex bias (Table 2). Human studies have demonstrated that female responses to heat acclimation may differ from those of male participants (Avellini et al., 1980; Mee et al., 2015). In addition, we previously reported that sex differences are associated with the risk of exertional heat illness during horse racing (Takahashi & Takahashi, 2020), and it is possible that sex differences affect heat adaptation in horses and humans. However, Mee et al. (2015) showed that long‐term heat acclimation (10 sessions over 17 days) induced a similar adaptive response, including a reduction in rectal temperature and improved exercise capacity in both males and females (Mee et al., 2015). Although there is limited information on sex differences in adaptation to heat stress in Thoroughbred horses, we previously reported that there were no sex differences in the mRNA levels of HSPs and mitochondria‐related proteins after acute exercise in hot conditions (Ebisuda, Mukai, Takahashi, Yoshida, et al., 2023). Therefore, we considered the sex bias in this study to be minimal and limited.

## 5 CONCLUSION

We investigated the effect of heat acclimation (three sessions/week for three weeks) on physiological parameters and proteins associated with heat stress and mitochondrial biogenesis in Thoroughbred skeletal muscles. We demonstrated that repeated heat exposure combined with high‐intensity exercise elicited improvements in exercise performance and thermoregulatory responses under hot conditions and protective adaptation to heat stress in skeletal muscles. Therefore, heat acclimation or acclimatization may be a promising training strategy for Thoroughbred racehorses to reduce the adverse effects of heat stress, including exertional heat illness, and contribute greatly to equine welfare.

## AUTHOR CONTRIBUTIONS

YE, KM, YT, and HO conceived and designed the study. YE, KM, YT, TY, AK, TM, HM, and HO performed the experiments. YE, KM, YT, TY, and HO collected the data. YE, AK, TM, and HM analyzed the data. YE wrote the first draft of the manuscript. All authors contributed to the manuscript revision and have read and approved the submitted version.

## FUNDING INFORMATION

The research was funded by the Japan Racing Association.

## CONFLICT OF INTEREST STATEMENT

This study was funded by the Japan Racing Association. YE, KM, YT, TY, and HO are employees of the Japan Racing Association.

## Data Availability

The data that support the findings of this study are available from the corresponding author upon reasonable request.
